# Determinants of Poor Glycemic Control in Patients with Kidney Transplants: A Single-Center Retrospective Cohort Study in Canada

**DOI:** 10.1177/2054358120922628

**Published:** 2020-05-18

**Authors:** Daniel Chan Chun Kong, Ayub Akbari, Janine Malcolm, Mary-Anne Doyle, Stephanie Hoar

**Affiliations:** 1 Faculty of Medicine, University of Ottawa, ON, Canada; 2 Division of Nephrology, The Ottawa Hospital, ON, Canada; 3 Division of Endocrinology, The Ottawa Hospital, ON, Canada

**Keywords:** kidney transplantation, diabetes mellitus, new-onset diabetes after transplant, glycemic control, risk factors

## Abstract

**Background:**

Kidney transplant immunosuppressive medications are known to impair glucose metabolism, causing worsened glycemic control in patients with pre-transplant diabetes mellitus (PrTDM) and new onset of diabetes after transplant (NODAT).

**Objectives:**

To determine the incidence, risk factors, and outcomes of both PrTDM and NODAT patients.

**Design:**

This is a single-center retrospective observational cohort study.

**Setting:**

The Ottawa Hospital, Ontario, Canada.

**Participant:**

A total of 132 adult (>18 years) kidney transplant patients from 2013 to 2015 were retrospectively followed 3 years post-transplant.

**Measurements:**

Patient characteristics, transplant information, pre- and post-transplant HbA1C and random glucose, follow-up appointments, complications, and readmissions.

**Methods:**

We looked at the prevalence of poor glycemic control (HbA1c >8.5%) in the PrTDM group before and after transplant and compared the prevalence, follow-up appointments, and rate of complications and readmission rates in both the PrTDM and NODAT groups. We determined the risk factors of developing poor glycemic control in PrTDM patients and NODAT. Student *t*-test was used to compare means, chi-squared test was used to compare percentages, and univariate analysis to determine risk factors was performed by logistical regression.

**Results:**

A total of 42 patients (31.8%) had PrTDM and 12 patients (13.3%) developed NODAT. Poor glycemic control (HbA1c >8.5%) was more prevalent in the PrTDM (76.4%) patients compared to those with NODAT (16.7%; *P* < .01). PrTDM patients were more likely to receive follow-up with an endocrinologist (*P* < .01) and diabetes nurse (*P* < .01) compared to those with NODAT. There were no differences in the complication and readmission rates for PrTDM and NODAT patients. Receiving a transplant from a deceased donor was associated with having poor glycemic control, odds ratio (OR) = 3.34, confidence interval (CI = 1.08, 10.4), *P* = .04. Both patient age, OR = 1.07, CI (1.02, 1.3), *P* < .01, and peritoneal dialysis prior to transplant, OR = 4.57, CI (1.28, 16.3), *P* = .02, were associated with NODAT.

**Limitations:**

Our study was limited by our small sample size. We also could not account for any diabetes screening performed outside of our center or follow-up appointments with family physicians or community endocrinologists.

**Conclusion:**

Poor glycemic control is common in the kidney transplant population. Glycemic targets for patients with PrTDM are not being met in our center and our study highlights the gap in the literature focusing on the prevalence and outcomes of poor glycemic control in these patients. Closer follow-up and attention may be needed for those who are at risk for worse glycemic control, which include older patients, those who received a deceased donor kidney, and/or prior peritoneal dialysis.

## Abrégé

**Contexte:**

Les médicaments immunosuppresseurs prescrits à la suite d’une transplantation rénale sont connus pour altérer le métabolisme du glucose, rendant plus difficile le contrôle de la glycémie chez les patients diabétiques avant l’intervention (DbAvT — diabétiques avant la transplantation) et chez les patients devenus diabétiques après l’intervention (NDbApT — nouveaux diabétiques après la transplantation).

**Objectif:**

Déterminer l’incidence d’un contrôle de la glycémie déficient, les facteurs de risque et les résultats chez les patients DbAvT et NDbApT.

**Type d’étude:**

Il s’agit d’une étude de cohorte rétrospective et observationnelle qui s’est tenue dans un seul center.

**Cadre:**

L’hôpital d’Ottawa (Ontario), au Canada.

**Sujets:**

Les adultes receveurs d’une greffe rénale entre 2013 et 2015 (n=132) ont été suivis rétrospectivement sur une période de trois ans post-transplantation.

**Mesures:**

Les caractéristiques des patients, les informations relatives à la greffe, les taux d’HbA1C et la glycémie pré- et post-transplantation, les rendez-vous de suivi, les complications et les réadmissions.

**Méthodologie:**

Nous nous sommes d’abord penchés sur la prévalence d’un contrôle glycémique déficient (HbA1c >8,5 %) dans le groupe DbAvT avant et après la greffe, puis nous avons comparé la prévalence, les rendez-vous de suivi et les taux de complications et de réadmission pour les deux groupes. Nous avons déterminé les facteurs de risque d’un mauvais contrôle glycémique chez les patients DbAvT et NDbApT. Les moyennes ont été comparées à l’aide du test t de Student, et le test du chi carré a servi à comparer les pourcentages. L’analyze univariée pour déterminer les facteurs de risque a été effectuée par régression logistique.

**Résultats:**

Parmi les 132 patients étudiés, 42 (31,8 %) étaient DbAvT et 12 (13,3 %) le sont devenus après l’intervention (NDbApT). La prévalence d’un mauvais contrôle de la glycémie (HbA1c >8,5 %) était plus élevée chez les patients DbAvT que chez les patients NDbApT (76,4 % contre 16,7 %; p<0,01). Les patients DbAvT étaient plus susceptibles d’être suivis par un endocrinologue (p<0.01) et une infirmière spécialisée en diabète (p<0.01) comparativement aux patients NDbApT. Aucune différence n’a été observée entre les deux groupes pour les taux de complications et de réadmission. Un greffon provenant d’un donneur décédé a été associé à un contrôle glycémique déficient (RC=3,34; IC 95 :1,08-10,4; p=0,04). Le développement d’un NDbApT a été associé à la fois à l’âge du patient (RC=1,07 IC 95: 1,02-1,3; p<0,01) et à un traitement de dialyze péritonéale (OR=4,57; IC 95: 1,28-16,3; p=0,02) avant la greffe.

**Limites:**

Nos résultats sont limités par la faible taille de l’échantillon. Nous n’avons pu rendre compte des dépistages effectués hors de notre center ni des rendez-vous de suivi avec un médecin de famille ou un endocrinologue dans la communauté.

**Conclusion:**

Un contrôle glycémique déficient est fréquent chez les patients greffés d’un rein. Les cibles glycémiques des patients DbAvT ne sont pas rencontrées dans notre center et notre étude met en lumière les lacunes de la littérature sur la prévalence et les résultats d’un mauvais contrôle glycémique chez ces patients. Un suivi plus étroit et une plus grande attention pourraient être nécessaires pour les patients susceptibles de voir leur contrôle glycémique se détériorer, notamment les personnes âgées, les receveurs d’un rein provenant d’un donneur décédé et les patients traités par dialyze péritonéale avant l’intervention.

## What was known before

New onset of diabetes after kidney transplantation (NODAT) is a common complication, and its incidence, associated risk factors, and adverse outcomes have been well studied in the literature. However, there exist very few Canadian studies on this topic and even fewer studies that have looked at the impact of kidney transplant immunosuppressive medications on the glycemic control of patients with pre-existing diabetes (PrTDM).

## What this adds

Our study adds to the limited Canadian research on the incidence, risk factors, and adverse outcomes of both NODAT and PrTDM population.

## Introduction

Kidney transplantation is the preferred treatment for patients with end-stage renal disease (ESRD), due to its cost effectiveness and ability to provide a better quality of life and prognosis than maintenance dialysis.^1-3^ In the kidney transplant population, diabetes mellitus is an extremely common disease.^4^ Immunosuppressive medications taken post-transplant (eg, cyclosporine, tacrolimus, prednisone) are known to impair glucose metabolism,^5^ leading to both worsened glycemic control in patients with pre-transplant diabetes mellitus (PrTDM) and new onset of diabetes after kidney transplant (NODAT). Diabetes in the kidney transplant population, whether pre-existing or newly acquired, is associated with increased risk for complications, including increased risk for infection, graft failure, cardiovascular complications, and increased mortality.^6-11^

New onset of diabetes after kidney transplant has been well studied in the literature. The reported incidence of NODAT is broad ranging from 10% to 74% depending on varying diagnostic criteria, screening protocols, and follow-up duration.^12^ Risk factors that are associated with NODAT include age, body mass index (BMI), African-American race, pre-diabetes prior to transplant, prednisone dose, tacrolimus use, receiving a deceased donor kidney, and cytomegalovirus (CMV) and hepatitis C virus infections.^12^

In Canada, the data on the incidence and risk factors of NODAT are limited. The few studies that exist have primarily investigated the incidence and risk factors associated with poor glycemic control in kidney transplant patients with PrTDM. The aim of this single-center study is to determine the incidence, risk factors, and outcomes of both poor glycemic control post-transplant and NODAT in this population, as well as identify any gaps in their care.

## Methods

This is a retrospective analysis of kidney transplant patients treated at a single Canadian tertiary center (The Ottawa Hospital) between January 9, 2013 and January 9, 2015. Data were collected until 3 years post-transplant for each subject and were collected via electronic medical record and paper charts. There were no exclusion criteria as part of this review.

The data collected were divided into 3 categories: (1) baseline/pre-transplant (2) during hospital stay post-transplant, and (3) 3 years post-transplant. Baseline data included age, sex, race, BMI, blood pressure at transplant, etiology of ESRD, type of renal replacements therapy prior to transplant, comorbidities, donor status (living vs deceased, donor age), and medications prior to transplant. In hospital data included days in hospital, type of immunosuppressive agents used, total dose of prednisone, days on intravenous insulin, and number of in-hospital complications. Follow-up data included follow-up appointments with members of the diabetes health care team and number of complications and rehospitalizations during the 3-years post-transplant. Random blood glucose, HbA1c, creatinine, albumin-to-creatinine ratio (ACR), and serum concentrations of immunosuppressive agents were collected quarterly from routine blood tests during the 3 years following transplant.

This study was approved by the Ottawa Health Science Network Research Ethics Board.

### Definitions

Pre-transplant diabetes mellitus was defined as the presence of a confirmed diagnosis of diabetes prior to transplantation as documented in the pre-transplant evaluation note or transplant surgery admission note. New onset of diabetes after kidney transplant was defined by at least one of the following criteria: (1) a random glucose >11.1 mmol/L, (2) a HbA1c >6.5%, or (3) the prescription of a new diabetes medication in patients without a previous diagnosis of diabetes. These criteria were based on the guidelines from the 2013 International Consensus Meeting on Post-transplantation Diabetes Mellitus.^13^ *Poor glycemic control* was defined as having at least one HbA1c >8.5% based off less stringent cut offs from the American Diabetes Association,^14^ with *adequate glycemic control* defined as HbA1c <8.5%. *Very poor glycemic control* was defined as having at least HbA1c >10% as this has been associated with random glucose levels >12 mmol/L.^15^

Complications in hospital included wound issues, urinary tract infections (UTI), pneumonia, cardiac events, rejection, anemia requiring transfusion, and delayed graft function. Cardiac events included coronary artery disease, stroke, and severe peripheral arterial occlusive disease. Delayed graft function was defined as a requirement of hemodialysis post-transplant during hospital admission.

Post-transplant complications included UTI, rejection, and CMV infection that were determined by urine cultures, renal biopsy, and CMV DNA titers respectively.

Elevated serum levels of tacrolimus were considered >8 ng/mL at 3 months and >6 ng/mL after 6 months post transplant. Elevated serum cyclosporine was defined as >800 ng/mL at 3 months and >600 ng/mL after 6 months post-transplant. Elevated blood glucose levels in hospital were defined as having a point of care glucose level of >11 mmol/L.

### Statistical Analysis

Continuous variables were expressed as means with standard deviations. Categorical variables were expressed as numbers and percentages. Student *t*-test was used to compare means, and chi-squared test was used to compare percentages. To determine whether both poor glycemic control was associated with adverse outcomes, and risk factors for poor glycemic control and NODAT, a univariate analysis was performed by logistical regression. We used one-way analysis of variance (ANOVA) to assess relationship between HbA1C and time post transplantation. For all tests, a *P* value, less or equal to .05, was considered to indicate statistical significance. All statistical analyses were computed with MedCalc for Windows (version 19.0; MedCalc Software, Ostend, Belgium).

## Results

### General Characteristics

There were a total of 170 kidney transplants from 2013 to 2015. Based on the availability of their paper charts, of these patients, 132 adult kidney transplant patients were included in our study, with 42 (31.8%) patients having PrTDM. The baseline data are displayed in Table 1.

**Table 1.** Demographics, Clinical, and Transplant Data of 132 Kidney Transplant Patients According to the Presence of Diabetes.

	Pre-KT diabetes (N = 42)	No diabetes (N = 90)	*P* value (95% CI)
Age—mean (*SD*)	53.4 (16.5)	47.6 (16.5)	.06 (‒11.9, 0.30)
Male (%)	61.9	58.8	.74 (‒14.8, 19.8)
Race—n (%)			
Caucasian	29 (69.0)	65 (72.0)	.72 (‒12.6, 20.2)
Black	4 (9.5)	7 (7.8)	.74 (‒7.7, 14.8)
Asian	4 (9.5)	5 (5.6)	.41 (‒5.0, 16.8)
Other	5 (11.9)	13 (14.4)	.70 (‒11.8, 13.5)
BMI at transplant (kg/m)—mean (*SD*)	28.6 (6.3)	26 (5.1)	*.01 (**‒4.64, 0.56**)
Blood pressure at transplant (mmHg)—mean (SD)	153/80 (23/11)	134/77 (21/12)	*SBP: <.01 (‒27.0, 11.0) DBP: .17 (‒7.32, 1.32)
Etiology of ESRD—n (%)			
Diabetic nephropathy	34 (81.0)	0 (0)	‒
Glomerulonephritis	4 (9.5)	36 (40.0)	‒
Polycystic kidney disease	0 (0)	20 (22.2)	‒
Ischemic nephropathy/hypertensive nephropathy	3 (7.1)	3 (3.3)	‒
Other	1 (2.4)	23 (25.6)	‒
Unknown	0 (0)	8 (8.9)	‒
Comorbidities—n (%)			
Hypertension	35 (83)	57 (63.3)	*.02 (**2.95, 33.0**)
Dyslipidemia	26 (61.9)	33 (36.7)	*.01 (**6.92, 41.2**)
Coronary artery disease	12 (28.6)	16 (17.8)	.1592 (‒3.84, 27.2)
Congestive heart failure	10 (23.8)	2 (2.2)	*.01 (**9.90, 36.4**)
Peripheral vascular disease	8 (19.1)	7 (7.8)	.06 (‒0.43, 26.1)
Type of renal replacement pre-transplant—n (%)			
Hemodialysis	25 (59.5)	51 (56.7)	.76 (‒15.1, 19.7)
Peritoneal dialysis	8 (19.0)	20 (22.2)	.68 (‒12.8, 16.4)
Preemptive	8 (19.0)	17 (18.9)	.99 (‒12.9, 15.9)
Donor status—n (%)			
Deceased donor	17 (40.5)	47 (52. 2)	.21 (‒6.46, 28.5)
Living donor	25 (59.5)	43 (47.8)	.21 (‒6.46, 28.4)
Donor age—n (%)			
<60 years	27 (64.3)	69 (76.7)	.14 (‒3.61, 29.3)
>60 years	6 (14.3)	7 (7.8)	.25 (‒4.10, 20.6)
Immunosuppression—n (%)			
Simulect	29 (69)	53 (58.9)	.27 (‒7.77, 25.9)
Thymoglobulin	16 (38)	35 (38.9)	.92 (‒16.9, 17.6)
Tacrolimus	30 (71.4)	79 (87.8)	*.02 (**2.26, 32.3**)
Cyclosporine	12 (28.5)	10 (11.1)	*.01 (**3.40, 33.2**)
Prednisone	39 (92.9)	87 (96.7)	.33 (‒3.80, 15.9)
Length of stay in hospital (days)—mean (SD)	8.2 (3.1)	8.9 (5.3)	.43 (‒1.04, 2.44)
3 months post-transplant			
Creatinine (mmol/L)—mean (SD)	125 (56)	128 (62)	.79 (‒19.2, 25.2)
eGFR (mL/min)—mean (SD)	61 (31)	60 (21)	.83 (‒10.1, 8.09)
ACR (mg/L)—mean (SD)	6.1 (7.2)	6.1 (8.0)	1.0 (‒2.87, 2.87)

*Note.* Demographic, clinical, and transplant data of 132 kidney transplant patients according to presence of diabetes. ESDR = end-stage renal disease; BMI = body mass index; SBP = systolic blood pressure; DBP = diastolic blood pressure; GFR = glomerular filtration rate; ACR = albumin creatinine ratio; SD = standard deviation; CI = confidence interval. *P ≤ 0.05.

Most of the patients were Caucasians and male. The mean age at transplantation was 53.4 years in the PrTDM group and 47.6 years in the no diabetes group. The leading cause of ESRD in the PrTDM patients was diabetic nephropathy (81%), and in those without diabetes was glomerulonephritis (40%). Of the 42 patients with PrTDM, 27 (64.3%) had type 2 diabetes, while the rest had type 1 diabetes. Body mass index (BMI; 28.6 vs 26, *P* = .01) and systolic blood pressure (153 vs 134, *P* < .01) at kidney transplant were significantly higher in patients with PrTDM. The most common comorbid conditions for both groups were hypertension, dyslipidemia, and coronary artery disease as determined by their physician. Hypertension (*P* = .02), dyslipidemia (*P* = .01), and congestive heart failure (*P* = .01) were more prevalent in the PrTDM group compared to those without diabetes. Hemodialysis was the most common form of renal replacement pre-transplant for both groups. Most of the kidney transplants were from donors who were younger than 60 years of age in both groups. A total of 17 PrTDM patients (40.5%) received a transplant from a deceased donor, which was comparable to the 47 recipients without diabetes (52.2%). Tacrolimus was the most common maintenance immunosuppression administered in both groups, but was significantly more commonly used in the nondiabetes group (*P* = .02), while cyclosporine was used most often in patients with PrTDM (*P* = .013; Table 1).

### Glycemic Control

Only 19 of the 42 PrTDM patients had a HbA1c measurement during the 6 months leading up to the transplant. Of these 19 patients, 9 (47.4%) had *poor glycemic control* and 2 (11.5%) had *very poor glycemic control*. HbA1c was measured consistently during the post-transplant period for each patient. Of the 42 patients with PrTDM, 30 (71.4%) had *poor glycemic control* and 13 (31%) had *very poor glycemic control* post-transplant. There was no significant difference between the proportion of poor glycemic control within 6 months before transplant vs within 3 years after transplant, although there was a trend toward an increase in proportion of patients with elevated HbA1c (Table 2).

**Table 2.** Glycemic Control Comparison Before and After Transplant in Patients With PrTDM.

(A) HbA1C control	n (%)	*P* value (95% CI)
(HbA1C >8.5%)		
Within 6 months pre-transplant (n = 19)	9 (47.4)	‒
Within 3 years post-transplant (n = 42)	30 (71.4)	.07 (‒1.74, 47.1)
(HbA1C >10%)		
Within 6 months pre-transplant (n = 19)	2 (11.5)	‒
Within 3 years post-transplant (n = 42)	13 (31.0)	.11 (‒4.70, 36.6)
(B) Random glucose (RG) control	Proportion RG >11 mmol/L (SD)	*P* value (95% CI)
Within 6 months pre-transplant (n = 41)	0.45 (0.42)	‒
Within 3 months post-transplant (n = 42)	0.41 (0.23)	.59 (‒0.19, 0.10)
3 months to 3 years post-transplant (n = 42)	0.46 (0.27)	.90 (‒0.14, 0.16)

*Note.* (A) Comparisons between proportions of patients with HbA1C readings >8.5% before vs after transplant. Same analysis performed for patients with HbA1C readings >10%.

(B) Comparisons between proportions of RG >11 mmol/L before transplant vs after 3 months and from 3 months to 3 years post-transplant, respectively. PrTDM = pre-transplant diabetes mellitus; SD = standard deviation; CI = confidence interval.

**Figure 1.**
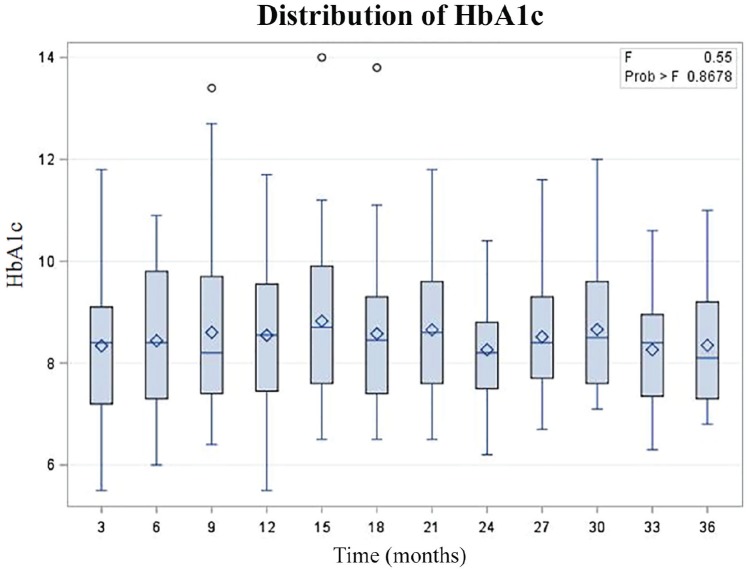
Relationship between HbA1C and time post-transplant.

ANOVA analysis revealed that that there was no statistically significant difference in HbA1c in the post-transplant period (Figure 1).

The proportion of random glucose concentrations >11 mmol/L were 45% at 6 months pre-transplant, 41% within the first 3 months post-transplant, and 46% from 3 months to 3 years post-transplant (Table 2).

The incidence of NODAT was 13.3% (total of 12 patients). Poor glycemic control was more prevalent in the PrTDM patients compared to those with NODAT 3 months to 3 years post-transplant (*P* < .01; Table 3).

**Table 3.** Comparison of Poor Glycemic Control in the PrTDM and NODAT Population Post-Transplant (Poor Glycemic Control Defined as HbA1C > 8.5% at Any Point During Study).

	n (%)	*P* value (95% CI)
PrTDM patients with poor glycemic control (n = 42)	30 (71.4)	‒
NODAT patients with poor glycemic control (n = 12)	2 (16.7)	*<.01 (**22.8, 71.3**)

*Note.* Comparison of the proportions of poor glycemic control in the PrTDM vs NODAT population following transplant. PrTDM = pre-transplant diabetes mellitus; NODAT = new onset of diabetes after transplant; CI = confidence interval. *P ≤ 0.05.

### Follow-Up with Health Care Team

PrTDM patients were more likely to receive follow-up with an endocrinologist compared to those with NODAT (PrTDM 76.2% vs NODAT 25%, *P* < .01). Similarly, 88% of PrTDM were seen by a diabetes nurse, compared to the 33.3% of NODAT patients (*P* < .01). All patients with NODAT and PrTDM received follow-up with a dietician, and more than 90% of patients from both groups were seen by a pharmacist (Table 4).

**Table 4.** Follow-Up by Health Care Professional According to Onset of Diabetes.

	PrTDM (N = 42)	NODAT (N = 12)	Difference (95% CI)	*P* value
Endocrinologist, n (%)	32 (76.2)	3 (25)	50.2 (19.4, 70.3)	*<.01
Diabetes nurse, n (%)	37 (88)	4 (33.3)	54.7 (24.1, 75.3)	*<.01
Pharmacist, n (%)	41 (97.6)	11 (92)	5.6 (−6.35, 32.7)	.37
Dietician, n %	42 (100)	12 (100)	‒	‒

*Note.* Comparison of the proportion of patients who received follow-up with members of the diabetes health care team between PrTDM vs NODAT patients. PrTDM = pre-transplant diabetes mellitus; NODAT = new onset of diabetes after transplant; CI = confidence interval. *P ≤ 0.05.

### Complications and Rehospitalizations

Patients with both PrTDM and NODAT did not have more rehospitalizations or increased prevalence of complications during initial hospitalization and after discharge compared to those without diabetes (Table 5). Similarly, patients with poor glycemic control (HbA1c >8.5%) did not have a greater risk for rehospitalizations and complications compared to those with adequate glycemic control (Table 6) from 3 months to 3 years post-transplant.

**Table 5.** Poor Outcomes in the Kidney Transplant Population.

	Complications in hospital		Readmissions		Complications post-transplant	
	n (%)	*P* value (95% CI)	n (%)	*P* value (95% CI)	n (%)	*P* value (95% CI)
No diabetes (n = 78)	13 (16.7)	‒	32 (41.0)	‒	40 (51.3)	‒
PrTDM (n = 42)	6 (14.3)	.73 (‒12.7, 14.8)	22 (52.4)	.23 (‒6.98, 29.0)	24 (57.1)	.29 (‒11.3, 41.9)
NODAT (n = 12)	2 (16.7)	>.99 (‒15.5, 28.9)	7 (58.3)	.26 (‒11.3, 41.9)	5 (41.7)	.54 (‒18.9, 34.3)

*Note.* Comparison between the number of complications during hospital admission, readmissions, and complications 3 months to 3 years post-transplant between patients without diabetes vs those with pre-transplant diabetes mellitus (PrTDM) and NODAT, respectively. NODAT = new onset of diabetes after transplant; CI = confidence interval.

**Table 6.** Risk of Developing Adverse Outcomes in Patients with Poor Glycemic Control (HbA1c >8.5).

	OR (95% CI)	*P* value
Complications in hospital	1.15 (0.65, 2.03)	.63
Readmission	0.97 (0.64, 1.49)	.91
Complications post-transplant	1.04 (0.68, 1.60)	.85

*Note.* Determining whether poor glycemic control increase risk of developing complications in hospital, being readmitted to hospital, and having post-transplant complications from 3 months to 3 years post-transplant. OR = odds ratio; CI = confidence interval.

### Risk Factors for Poor Glycemic Control in PrTDM and Risk Factors for Developing NODAT

Results from the univariate logistical regression showed that receiving a transplant from a deceased donor was associated with having poor glycemic control, OR = 3.34, CI (1.08, 10.4), *P* = .04 (Table 7). Both patient age, OR = 1.07, CI (1.02, 1.3), *P* < .01, and peritoneal dialysis prior to transplant, OR = 4.57, CI (1.28, 16.3), *P* = .02, were associated with NODAT (Table 8).

**Table 7.** Risk Factors for Poor Glycemic Control in Patients With Diabetes.

	OR (95% CI)	*P* value
Age	0.99 (0.95, 1.03)	.64
Weight	0.99 (0.97, 1.02)	.59
BMI	0.97 (0.87, 1.09)	.63
Family history of diabetes	1.12 (0.36, 3.53)	.85
Type of dialysis—hemodialysis	0.79 (0.26, 2.35)	.67
Type of dialysis—peritoneal dialysis	0.89 (0.26, 3.05)	.85
Type of donor (*deceased donor, living donor)	3.34 (1.08, 10.4)	*.04
Donor age	0.99 (0.95, 1.03)	.75
Elevated tacrolimus levels or cyclosporine levels after 3 months post-transplant	0.33 (0.04, 3.21)	.34
Prednisone dose during hospital	1.0 (0.99, 1.0)	.09
High glucoscans (>11.0 mmol/L) during hospital (no. of high days)	1.0 (0.86, 1.17)	.96
Days on IV insulin in hospital	1.25 (0.89, 1.77)	.2
Delayed graft function	0.49 (0.14, 1.75)	.27
Rejection	1.17 (0.25, 5.51)	.84
eGFR at 3 months	1.01 (0.99, 1.03)	.39
Urine ACR at 3 months	1.03 (0.95, 1.11)	.45
Follow-up with endocrinologist	1.28 (0.26, 6.24)	>.99

*Note.* Risk factors for poor glycemic control (HbA1c >8.5%) in patients with pre-transplant diabetes. OR = odds ratio; CI = confidence interval; BMI = body mass index; GFR = glomerular filtration rate; IV = intravenous; ACR = albumin creatinine ratio. *P ≤ 0.05.

**Table 8.** Risk Factors for NODAT.

	OR (CI)	*P* value
Age	1.07 (1.02, 1.3)	*<.01
Weight	1.01 (0.98, 1.05)	.5
BMI	1.11 (0.96, 1.29)	.15
Family history of diabetes	1.29 (0.39, 4.28)	.68
Type of dialysis—hemodialysis	0.50 (0.45, 1.7)	.26
Type of dialysis—peritoneal dialysis	4.57 (1.28, 16.3)	*.02
Type of donor (deceased donor, living donor)	0.50 (0.14, 1.80)	.29
Donor age	0.99 (0.95, 1.03)	.68
Elevated tacrolimus levels or cyclosporin levels after 3 months post-transplant	1.0 (0.20, 5.11)	1.0
Prednisone dose during hospital	1.0 (0.99, 1.0)	.97
High glucoscans during hospital (no. of high days)	1.55 (0.87, 2.74)	.14
Days on IV insulin in hospital		
Delayed graft function	0.91 (0.18, 4.64)	.91
Rejection	1.10 (0.12, 9.94)	.94
eGFR at 3 months	0.99 (0.97, 1.03)	.96
Urine ACR at 3 months	1.02 (0.96, 1.09)	.5

*Note.* Risk factors for NODAT. NODAT = new onset of diabetes after transplant; OR = odds ratio; CI = confidence interval; BMI = body mass index; IV = intravenous. *P ≤ 0.05.

## Discussion

### Glycemic Control

The analysis of this single-center retrospective cohort study has generated several important findings, with the most relevant being the inadequate glycemic control seen in the PrTDM patients pre- and post-transplant. Almost 3 quarters of the patients with PrTDM had poor glycemic control, with half of these patients having an HbA1c reading >10% post-transplant. The literature primarily focuses on NODAT, and few studies have looked at the prevalence and outcomes of poor glycemic control in the kidney transplant population. One study, by Taber et al,^9^ reported that 51.9% of the PrTDM had poor glycemic control, defined as an average HbA1C >7% or fasting glucose of ≥8.3 mmol/L. In terms of outcomes, Tabet et al^9^ study showed that there was no difference in mortality and graft loss between transplant patients with uncontrolled diabetes compared to those with controlled diabetes, although it is possible that a difference would have been observed had the authors used a higher HbA1c cut-off in their definition for poor glycemic control.

Patients with diabetes often have multiple comorbidities. The presence of diabetes in patients undergoing kidney transplant increases the likelihood to develop complications such as infections,^7,10,16^ cardiovascular events,^7,9,10,16,17^ and increased risk for mortality.^6,8,9^ Graft dysfunction^9,18-20^ is also more likely in the PrTDM population, which is supported by the findings in our study. While it is reasonable to assume that PrTDM patients with poorer glycemic control are more likely to have more complications and poorer outcomes, our study did not support this hypothesis. There is currently a paucity of observational data looking at longer term outcomes in this patient population, representing an important area to focus for future research.

Different immunosuppressants are known to affect glycemic control in patients, with tacrolimus being shown to be associated with greater prevalence of post-transplant hyperglycemia and greater incidence of NODAT when compared to cyclosporine.^21-25^ As per standard of care, our study found that patients with PrTDM were less likely to receive tacrolimus and more likely to receive cyclosporine. However, despite being treated with a more glycemic-friendly immunosuppressant, a large proportion of patients still had poor glycemic control.

### Incidence of NODAT

In the current study, the overall incidence of NODAT 3 years post-transplant was slightly higher than that of another single-center Canadian study with similar diagnostic criteria.^26^ Although both studies indicated a fairly high incidence, many recent studies have reported even higher incidences 3 years post-transplant, such as those reported in the United States (16%-24%),^10,27^ Europe (17%-30%),^28-30^ and Asia (31%-34%).^16,31^ Differences between studies may be due to variability in NODAT diagnostic criteria and patient demographics such as BMI and ethnicity, as well as post-transplant surveillance.^26^

### Follow-Up Appointments with Health Care Team

We found that patients with PrTDM were also more likely to be seen by both an endocrinologist and diabetes nurses compared to those with NODAT. This is possibly due to the fact that PrTDM patients are able to readily schedule follow-up appointments with an already established diabetes health care team. Nonetheless, there were a large number of PrTDM patients that were not followed at our center by the diabetes team. In addition, despite having more follow-up compared to those with NODAT, PrTDM patients still showed a higher prevalence of poor glycemic control after transplant. These findings highlight that follow-up appointments with the PrTDM patients remain an important area needing more attention and an opportunity to improve outcomes in this patient population.

### Complications and Rehospitalizations

The current study demonstrated that patients with PrTDM and NODAT were not more likely to have rehospitalization or complications compared to patients without diabetes. There are currently conflicting studies associating NODAT as a risk factor for rehospitalization;^32,33^ however, PrTDM has been well supported by the literature as a risk factor for hospital readmission with most studies showing high rates of rehospitalization within the first year after transplant.^18,34,35^ With post-transplant rehospitalization representing a main contributor to health care expenses after kidney transplant,^33-37^ future studies are needing in clarifying whether poor glycemic control predisposes them to adverse outcomes, which may support the need for preventative strategies, as potential means for resource optimization.

### Risk Factors

Receiving a kidney from a deceased donor is a well-known risk factor for NODAT.^12,26,38,39^ However, to our knowledge, no other study has reported a deceased donor kidney to be a significant risk for poor glycemic control in patients with PrTDM, although the mechanism is likely the same. Deceased donor kidney allografts have more ischemia and are more likely to come from older and more comorbid donors. They are known to express higher levels of proinflammatory cytokines in comparison to living kidney donor grafts, whether related or unrelated, demonstrating that graft injury is more likely with a deceased donor kidney.^40-42^ The resulting increased risk of rejection potentially requires more aggressive immunosuppression, thus further impairing glycemic control. In addition, higher levels of systemic inflammation are known to play a major role in the pathogenesis of type 2 diabetes,^43^ which would be expected to be seen from deceased donor recipients. To note, however, our study did not find donor age, graft rejection, in-hospital prednisone dose, and high serum level of tacrolimus or cyclosporine post-transplant to be independent risk factors for poor glycemic control in patients with PrTDM.

Risk factors for NODAT were age and prior peritoneal dialysis. Just as with type 2 diabetes, NODAT is more likely to occur in older patients.^12,27,44,45^ Insulin resistance that develops with aging is mainly attributed to factors that include diminished physical activity, poor diet, obesity, and loss of lean body mass. There is also the development of a functional decline of pancreatic B-cells as well as impaired B-cell adaptation to insulin resistance, resulting in glucose intolerance.^46^

Our study demonstrated peritoneal dialysis as a risk factor for NODAT. Our results are similar to a cohort study by Madziarska and colleagues of 377 patients, who were the first to report a significantly greater incidence of NODAT in peritoneal dialysis patients (35.4%), when compared to those who received hemodialysis (21.2%).^47^ However, a study by Courivaud and colleagues found no impact of pre-transplant dialysis modality on incidence of NODAT.^48^ Peritoneal dialysis commonly involves infusing a dextrose dialysate into the peritoneal cavity to create an osmotic gradient for blood filtration and fluid exchange. This poses a risk for hyperglycemia due to the high glucose load from the dialysate; this has been shown to contribute to impaired fasting glucose and metabolic syndrome,^49^ predisposing patients for NODAT. Our study did not find that BMI had a significant effect on the development of NODAT, despite most studies demonstrating it as an independent risk factor for NODAT.^50^

### Limitations

Several limitations of this study are worth noting. First, the small sample size limits this study from performing multivariate analysis to control for confounding variables. In addition, the retrospective cohort design of our study does not allow us to establish a causal relationship between many independent variables and our main outcome, glycemic control. This study also could not account for any diabetes screening performed outside of the Ottawa Hospital or follow-up with family physicians or community endocrinologists. This is primarily due to a lack of access to patient charts and electronic medical records in the community, which may potentially underestimate the incidences of NODAT as well as follow-ups with members of the diabetes health care team. Also, of note, our study was limited by the available HbA1c readings pre-transplant for those with PrTDM, with 54.8% (23/42) of them having absent readings. We also did not perform standard oral glucose testing and had to rely on random glucose and HbA1c measurement alone to classify NODAT and poor glycemic control. This approach primarily reflects the practice of most centers, given that oral glucose testing is a cumbersome test. In addition, our study did not do multivariable analysis and there could be collinearity between our identified risk factors (age, diseased donor, and peritoneal dialysis). This study was also performed primarily on Caucasians (71.2%), and the results may not be applicable to other ethnicities. Finally, another potential limitation to consider is the use of HbA1c as a measure of glycemic control in this patient population. Poor renal function has been shown to be associated with a skewed HbA1c as a result of the alteration in the hemoglobin lifespan and the administration of erythropoietin.^51-53^ Therefore, the diagnosis for poor glycemic control and NODAT using HbA1c is likely underestimated, particularly as the estimated glomerular filtration rate (eGFR) was not normalized. However, the unreliability of HbA1c is typically only significant in moderate to severe renal impairment (eGFR <45 mL/min/1.73 m^2^).^54^ As the patients in our study had good renal function post-transplant with eGFR ≥ 60 mL/min/1.73 m^2^ (Table 1), we expect that the impact of renal function on HbA1c reliability is likely not clinically significant. For future studies, emerging potential markers of glycemic control for patients with ESRD that can be explored include glycated albumin^52^ and fructosamine;^55^ however, their use as more accurate alternatives to HbA1c in this patient population is still unclear.^56^

## Conclusions

In conclusion, poor glycemic control is a common and important issue in the kidney transplant population. The findings of this study indicate that glycemic targets for patients with PrTDM are not being met and highlights the gap in the literature focusing on the prevalence and outcomes of poor glycemic control in these patients. Significant risk factors for NODAT were age and previous peritoneal dialysis, and a risk factor for poor glycemic control was type of donor. Our study adds to the body of knowledge for these risk factors, and their recognition in this patient population may be useful to prevent a deterioration in glycemic control. Overall, early identification of impaired glycemic control in the post kidney transplant population will allow implementation of lifestyle modifications, adjustments of immunosuppressive, and initiation of glucose-lowering therapies as indicated, thereby preventing progression of diabetes or NODAT and associated complications.
